# Lionfish misidentification circumvents an optimized escape response by prey

**DOI:** 10.1093/conphys/cow064

**Published:** 2016-12-15

**Authors:** Mark I. McCormick, Bridie J. M. Allan

**Affiliations:** 1 ARC Centre of Excellence for Coral Reef Studies, James Cook University, Townsville, QLD 4811, Australia; 2 Department of Marine Biology and Aquaculture, James Cook University, Townsville, QLD 4811, Australia

**Keywords:** Chemical alarm cue, coral reef fishes, escape response, predator–prey, *Pterois volitans*, risk assessment

## Abstract

Invasive lionfish represent an unprecedented problem in the Caribbean basin, where they are causing major changes to foodwebs and habitats through their generalized predation on fishes and invertebrates. To ascertain what makes the red lionfish (*Pterois volitans*) such a formidable predator, we examined the reaction of a native damselfish prey, the whitetail damsel (*Pomacentrus chrysurus*), to a repeatable startle stimulus once they had been forewarned of the sight or smell of lionfish. Fast-start responses were compared with prey forewarned of a predatory rockcod (*Cephalopholis microprion*), a corallivorous butterflyfish (*Chaetodon trifasctiatus*) and experimental controls. Forewarning of the sight, smell or a combination of the two cues from a rockcod led to reduced escape latencies and higher response distances, speed and maximal speed compared with controls, suggesting that forewarning primed the prey and enabled a more effective escape response. In contrast, forewarning of lionfish did not affect the fast-start kinematics measured, which were the same as in the control and non-predatory butterflyfish treatments. Lionfish appear to be able to circumvent mechanisms commonly used by prey to identify predators and were misclassified as non-predatory, and this is likely to contribute to their success as predators.

## Introduction

The invasive red lionfish (*Pterois volitans*) has been described as one of the greatest threats to the Caribbean marine ecosystem. Released by accident or misadventure in the mid-1980s off the coast of Florida, they have spread throughout the Caribbean, where populations have grown exponentially, with substantial impact to the community composition (Betancur-R *et al*., 2011; Albins and Hixon, 2013; Côté *et al*., 2013; Benkwitt, 2014; Ballew *et al*., 2016). Densities in some places of the Caribbean have been estimated at 400 ha^−1^ (Morris and Whitfield, 2009), with lionfish consuming 8–10% of their body weight per day (Green *et al*., 2011). To understand what contributes to the astonishing success of the lionfish, it is necessary to determine the way in which this predator interacts with its prey. Recently, research has found that lionfish can circumvent the general mechanism by which prey rapidly learn about predators (Lönnstedt and McCormick, 2013), known as associative learning. This learning mechanism is critical for the survival of vulnerable juveniles (Lönnstedt *et al*., 2012), and bypassing this method of predator identification may be key to the lionfish attaining one of the highest strike success rates of any piscivore (85%; Green *et al*., 2011). It is very difficult to determine the aspects of the predator–prey interaction that make this invasive species so successful in the Caribbean without understanding how prey interact with lionfish within their native range. It is currently unclear how lionfish manage to avoid being labelled as a threat, whether it is generally applicable to other fish prey species, and at what stage in the predator–prey encounter this disruption in learning occurs.

When any prey is confronted with a predator, it must attempt to evade capture. As failure results in damage or death, there is a strong selective pressure to have well-developed means of escaping a predatory strike (Walker *et al*., 2005). The primary response by prey to a predatory attack is a fast-start response; an evasive manoeuvre designed to displace the prey out of the predator's strike range. Anaerobically fuelled and costly to undertake (Domenici and Blake, 1997), fast starts occur in a diverse group of taxa, including some invertebrates, fishes and amphibians (Bullock, 1984). Although there are economic arguments regarding exactly when to use a fast-start response (e.g. balancing vigilance with foraging; economic hypothesis; Ydenberg and Dill, 1986), the same arguments can be made with respect to the intensity of the response. The high cost of repaying oxygen debts from anaerobic metabolism (Moyes *et al*., 1993), the stress associated with chase anticipation (Boonstra, 2013), and disruption to other fitness-related activities suggest that it could be beneficial for animals to alter the intensity of their fast-start to match the intensity of the threat and, in so doing, minimize its cost.

Until recently, it was often assumed that the fast-start response was a largely autonomic response, with performance maximized by strong predator selection and little variation within individuals (Eaton and Hackett, 1984; Langerhans *et al*., 2004; Weiss *et al*., 2006). Other research on fishes has shown that escape performance is often below an individual's maximal performance (Ydenberg and Dill, 1986; Domenici, 2010) and that fast starts can show a high degree of within-individual variability (at times >60% coefficent of variation; Ramasamy *et al*., 2015); a situation some would suggest to be detrimental to survival (Walker *et al*., 2005). Recent studies have broken up the sequence of events that compose the escape response into parts that are under behavioural modification and those that are more autonomic (Marras *et al*., 2011; Allan *et al*., 2014), but this is probably best seen as a spectrum, with context influencing the location of the response variable. For instance, whether to respond and the time to react may be largely under some behavioural control, whereas physiological limitations on acceleration and maximal speed may make these traits more autonomic (Marras *et al*., 2011). A recent study found that forewarning of a predator, through either olfactory or visual cues, led to a marked increase in the fast-start performance for a juvenile fish (Ramasamy *et al*., 2015). This notion of ‘priming’ has also been observed with subthreshold responses to disturbance cues leading to suprathreshold responses to alarm cues (Ferrari *et al*. 2008; Vavrek *et al*. 2008). The high element of behavioural modification to fast-start responses suggests that the energetic costs are high and that it is worth optimizing despite the dire cost of underperformance.

Information to forewarn of the activity of predators in the vicinity of a prey often comes from the direct receipt of olfactory, visual or vibration cues. When a range of cues are available, aquatic prey often use smell as a key indicator of potential risk (Holmes and McCormick, 2011), while vision is used to determine the intentions of the predator and fine-tune the antipredator behaviour to minimize the cost of a response (McCormick and Manassa, 2008). The present study focuses on the part of the predator–prey sequence when the prey has become aware that a predator is in the vicinity and explores how prey then respond to a repeatable startle stimulus. Specifically, it examines whether the whitetail damselfish (*Pomacentrus chrysurus*) is able to alter its fast-start response when forewarned of a nearby red lionfish (*P. volitans*) compared with controls. Our prediction, based on the findings of Lönnstedt and McCormick (2013) and Ramasamy *et al*. (2015), was that cues from a predatory rockcod (*Cephalopholis microprion*) would enhance the fast-start performance of the damselfish above controls, whereas cues from a non-predator (the butterflyfish, *Chaetodon trifasciatus*) and predatory red lionfish would not. The present study gives further support to the intriguing hypothesis that lionfish somehow manage to circumvent associative learning, which has only to date been shown for the blue damselfish, *Chromis viridis* (Lönnstedt and McCormick, 2013). It is also only the second study to demonstrate that if prey can appropriately identify a predation risk they can optimize their escape response.

## Materials and methods

### Study species

The whitetail damselfish (Pomacentridae) is a rubble-associated planktivore commonly found across the Indo-Pacific (Allen, 1991). Newly metamorphosed whitetail damselfish were collected with light traps moored at least 50 m off the fringing reef of Lizard Island (14°40′12.13″S, 145°27′42.20″E), northern Great Barrier Reef, Australia. On the morning of capture, whitetail damselfish were transferred to 30 litre tanks and fed *Artemia* in excess of requirements twice daily. Juveniles were held for 5–7 days prior to use in experiments to allow recovery from the stress of capture. They were not fed for 12 h prior to commencement of the experimental trials to standardize for satiation. Salinity (35 ppt) and temperature (29°C) were kept constant throughout the study period and trials.

Two predator species provided chemical and visual cues for the experiment: the red lionfish (*P. volitans*; Scorpaenidae), a rare member of the fish assemblage around Lizard Island, and the common rockcod (*C. microprion*; Serranidae). The red lionfish naturally occurs broadly through the Pacific Ocean, whereas the rockcod has a Western Pacific distribution (Randall *et al*., 1997). Both naturally co-occur with whitetail damselfish. The common rockcod is known to be an unselective and important predator on newly settled and juvenile damselfishes (Holmes and McCormick, 2010), whereas the red lionfish is known to feed on juvenile damselfishes when available (e.g. Côté and Maljkovic, 2010; Cure *et al*., 2012). The former was caught using hand nets, whereas the latter was caught using underwater hook-and-line fishing. Responses of whitetail damselfish to the predator cues were compared with their response to cues from a common coral-eating non-piscivore, the three-lined butterflyfish (*C. trifasciatus*; Chaetodontidae), which was caught using hand nets and a barrier net. Adult fishes were given at least 5 days to recover from the stress of capture prior to their use in experiments. Predators were fed cardinalfish (*Apogon* sp.; Apogonidae) daily, which are phylogenetically distant from the focal damselfish. Butterflyfish were given some healthy hard coral (*Pocillopora damicornis*) to forage over. Tank experiments were undertaken at the Lizard Island research station.

### Conditioning treatment

As light-trap-caught fish are naïve to reef-based predators, it was necessary to standardize the prior history and experience with predators before the fast-start trials (Lönnstedt *et al*., 2012). Therefore, prior to trials commencing, juvenile whitetail damselfish (mean 14.5 mm standard length) were taught to recognize the predators as a threat by exposing them to the sight and odour of the predator at the same time as a damage-released chemical alarm cue from conspecifics. Chemical alarm cues were obtained through five superficial cuts to both sides of four euthanized (through cold shock) whitetail damselfish and each rinsed with 15 ml of seawater. This coupling of the visual or odour cues with a chemical alarm cue leads to the assignment of risk to the cues through a process known as associative learning (Suboski, 1990). To couple odour with the visual cue, lionfish or rockcod of similar sizes were put into transparent plastic bags full of aerated seawater and placed into each of the 12 whitetail damselfish holding tanks containing ~20 fish. Thirty millilitres of odour from the relevant predator (from their holding tank) and 15 ml of chemical alarm cue from whitetail damselfish were injected simultaneously into the individual holding tanks. Whitetail damselfish were conditioned to the butterflyfish in a similar way to that used for the predators; however, rather than the chemical alarm cue being added to the holding tanks, clean seawater was added. This should make the juveniles familiar with, but not scared of, the sight and odour of the butterflyfish. Odours from the predators (lionfish and rockcods) and non-predator (butterflyfish) were prepared by turning off the flowing seawater and leaving the tank containing the predators with aeration for 2 h. Prey were exposed to cues for 30 min following a standard protocol (Lönnstedt and McCormick, 2013). Whitetail damselfish were conditioned to butterflyfishes first, because this conditioning did not involve alarm cues. Fish were left for 2.5 h between conditioning events, during which time the tank was flushed with clean seawater. The sequence of rockcod and lionfish conditioning was alternated (six tanks had lionfish conditioning prior to rockcod, and six vice versa). Previous studies have shown that juvenile damselfish can learn visual or olfactory cues that represent danger after only one exposure when paired with an alarm cue (Ferrari *et al*., 2010), even if many novel cues are presented at the same time (Mitchell *et al.,* 2011b). Moreover, research shows that the cues are remembered for weeks after a single learning event (Brown and Smith, 1998; Mirza and Chivers, 2000). To reduce the impact of diet cues, adult cue source fishes (i.e. lionfish, rockcod and butterflyfish) were not fed within 12 h of being used in the conditioning trials or the fast-start arena (see below, ‘*Fast-start arena and protocol*’).

### Experimental treatments

The day after conditioning, the whitetail damselfish juveniles were carefully moved individually into a specially designed stimulus arena and given a 5 min acclimation period, by which time they moved freely around the circular arena. Afterwards, they were exposed to olfactory, visual or the combined cues from one of three adult fish species (of similar size: lionfish, rockcod or butterflyfish), plus a seawater injection (SW), and SW plus empty stimulus tank controls. The focal fish were then startled with a repeatable drop stimulus to elicit a fast start. Whitetail damselfish were only ever used once and were randomly allocated to one of the 11 treatment combinations.

### Fast-start arena and protocol

The fast-start arena consisted of a transparent circular arena (diameter 20 cm) within a 60 litre white-sided container (35 cm × 40 cm × 25 cm; Supplementary material Fig. S1) that had a Perspex bottom. Holes in the inner arena allowed flow of aerated water during acclimation. The water depth within the fast-start arena was 6 cm. This restricted movement in the vertical plane. The whole holding tank was illuminated by an LED light strip wrapped around the outside of the tank, with light penetrating with even illumination through the white plastic sides. Fast-start responses were elicited by the release of a tapered metal weight from above the water surface. This was accomplished by turning off an electromagnet to which the metal weight was attached. The metal weight was controlled by a piece of nylon line that was just long enough to allow the tapered tip to touch the surface of the water. In order to provide a sudden stimulation and allow calculation of the escape latency, the stimulus was released through a white polyvinyl chloride tube (diameter 40 mm, length 550 mm) suspended above the experimental arena, with the bottom edge at a distance of 10 mm above the water level. Fish were only startled when they moved to the middle portion of the tank, allowing an individual to move an equal distance in any direction and standardizing for fish position relative to the stimulus. Escape responses were recorded at 480 frames s^−1^ (Casio EX-ZR1000) as a silhouette from below, obtained by pointing the camera at a mirror angled at 45°. Prey escape variables were measured only when prey performed a C-start. A 1 cm line was drawn in the centre of the inner arena to enable calibration for video analysis. Trials were conducted between 08.00 and 16.00 h.

The experiment involved four cue sources (lionfish, rockcod, butterflyfish and disturbance control) crossed with three sensory cue types (olfactory, visual and olfactory with visual). Whitetail damselfish received either olfactory cues (fish cue or seawater) and/or visual cues (stimulus fish in adjacent tank or empty tank) prior to the startle stimulus (predator odours were prepared as described above). The seawater control consisted of 30 ml of water from the experimental arena re-injected into the inner tank through a tube attached to the stimulus weight tube that hung over the central arena (see Fig. S1). Predator or non-predator odour treatments involved the slow injection of 15 ml of seawater from the predator or non-predator holding tanks into the inner acclimation arena followed by 15 ml of seawater to ensure that the full cue was used. Visual cues were accomplished by placing each stimulus fish (one of three for each species) into a transparent 5 litre tank adjacent to the transparent stimulus arena. The visual stimulus for the control was an empty tank filled with seawater adjacent to the stimulus arena. Fish were left for an additional 5 min after the introduction of the odour or visual cues and then they were startled with the release of the stimulus weight once they had moved into the appropriate portion of the tank. The seawater in the experimental arena was changed after each trial to avoid a potential build-up of odours across trials.

### Kinematic variables

Kinematic variables associated with the fast-start response were analysed using the image-analysis software ImageJ, with a manual tracking plug-in (imagej.nih.gov/ij/). The centre of mass of each fish was tracked for the duration of the response. The following kinematic variables were measured.

- Response latency (in seconds) was measured as the time interval between the stimulus onset and the first detectable movement leading to the escape of the animal.
- Response distance (in metres) is a measure of the total distance covered by the fish during the first two flips of the tail (the first two axial bends, i.e. stages 1 and 2 defined based on Domenici and Blake, 1997), which is the period considered crucial for avoiding ambush predator attacks (Webb, 1976).
- Response speed (in metres per second) was measured as the distance covered within a fixed time (24 ms). This fixed duration was based on the average duration (22.8 ms) of stage 1 and 2 (as defined above).
- Maximal response speed (in metres per second) was measured as the maximal speed achieved at any time during stage 1 and stage 2.

### Statistical analyses

A two-factor multivariate analysis of variance (MANOVA) that included all four kinematic variables was undertaken to test whether there was a difference in the kinematic response of fish among ‘cue sources’ (levels: lionfish, rockcod, butterflyfish and control), ‘sensory modes’ (olfactory, visual, and olfactory with visual) and their interaction. Pillai's trace was chosen as the test statistic because it is robust to small sample sizes. The dependent variables were the four kinematic variables measured on each fish during their fast-start response. This was followed by ANOVAs and Fisher's least significant difference means comparisons to determine the nature of the significant difference found by MANOVA. The assumptions of normality and homogeneity of variance were examined with residual analysis. All variables were found to meet assumptions, with the exception of latency that was log10(*x*) transformed to improve normality. Type IV sums of squares were used owing to a missing control treatment combination (i.e. empty visual cue tank without SW injection).

## Results

**Figure 1:**
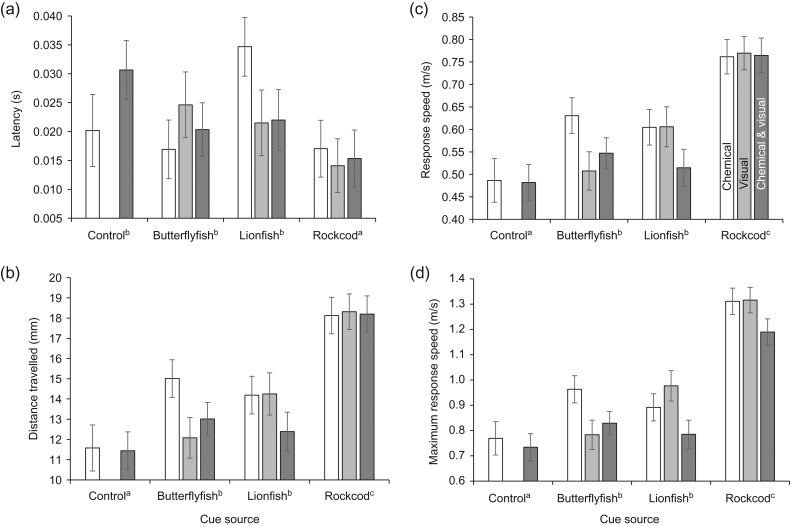
Comparison of the kinematics of a fast-start response of juvenile whitetail damselfish (*Pomacentrus chrysurus*) when forewarned of the presence of two predators (lionfish, *Pterois volitans*; and rockcod, *Cephalopholis microprion*), a non-predatory butterflyfish (*Chaetodon trifasciatus*) and controls (cue sources). Prey were exposed to the chemical scent of the adult fish species (open bars), their sight (light grey bars) or a combination of the two (dark grey bars). The kinematic variables measured were as follows: (**a**) latency (in seconds); (**b**) response distance (in millimetres); (**c**) response speed (in metres per second); and (**d**) maximal response speed (in metres per second). Means with standard errors are displayed. Letter superscripts on cue source labels are Fisher's least significant difference mean comparison groupings used to determine the nature of significant differences among cue sources.

A MANOVA found that the fast-start response of whitetail damselfish differed among cue sources (Pillai's trace = 0.52, *F* = 7.733, d.f. = 12,441, *P* < 0.0001), and these effects were not affected by the type of sensory mode (Pillai's trace = 0.06, *F* = 1.202, d.f. = 8, 292, *P* = 0.297), and the effect of cue source was consistent across sensory modes (interaction, Pillai's trace = 0.18, *F* = 1.420, d.f. = 20, 592, *P* = 0.105; Fig. 1). Latency to respond differed among cue sources (*F*_3,149_ = 6.690, *P* < 0.0003) but was unaffected by sensory mode (i.e. chemical, visual or chemical + visual; *F*_2,149_ = 0.853, *P* = 0.428) or the interaction between source and sensory mode (*F*_5,149_ = 2.234, *P* = 0.054). *Post hoc* tests showed that whitetail damselfish exposed prior to the startle stimulus with rockcod cues had significantly shorter latencies than those exposed to lionfish, butterflyfish or the controls (Fig. 1a).

Response distance, response speed and maximal response speed all showed the same trends and supported the pattern indicated by the MANOVA, with a significant difference among cue sources (*F*_3,152_ = 24.99, *P* < 0.0001; *F*_3,152_ = 24.17, *P* < 0.0001; *F*_3,152_ = 46.90, *P* < 0.0001, respectively), but no effect of sensory mode or interaction on the variables (Table 1). *Post hoc* tests indicated that the whitetail damselfish exposed to the controls had the lowest response, those exposed to the rockcod cues had the highest response, and those exposed to the lionfish and butterflyfish cues did not differ from one another in their response and were intermediate between the controls and rockcod (Fig. 1b, c and d).

**Table 1:** Factorial comparison of fast-start performance of whitetail damselfish when pre-exposed to one of four cue sources (control, butterflyfish, lionfish or rockcod) and three sensory modes (chemical, visual or chemical plus visual)

Variable	Cue source (3 d.f.)	Sensory mode (2 d.f.)	Cue source × sensory mode (6 d.f.)
Latency	6.69**	0.85	2.23
Response distance	24.99***	0.59	0.97
Response speed	24.17***	0.70	0.39
Maximal speed	46.90***	1.86	1.74

*F*-ratios and significance levels are given. **P = 0.0002; **P < 0.0001. Error d.f. 152 (except latency, with 149 d.f.).

## Discussion

The present study suggests that lionfish can circumvent the commonly used mechanisms of predator learning by prey, and this unique ability is likely to contribute to their success as an invasive predator. When whitetail damselfish prey were exposed to olfactory or visual cues from the predatory rockcod prior to being startled, they showed a heightened fast-start response, suggesting that forewarning of risk enhanced the efficacy of the burst response. This was in contrast to when cues of a non-predator were present, which did not affect the burst response compared with controls. Interestingly, the reaction by the prey to the lionfish cues was similar to the response to non-predators and controls: long response latencies and slower response speeds. This is despite having been taught that the lionfish represented a predatory threat using associative learning prior to the trial. This lack of a forewarning response means that prey have a much lower chance of eliciting a burst response that is optimal once a strike by a lionfish has been detected, reducing their likelihood of escape and potentially increasing mortality rates.

The forewarning effect found for whitetail damselfish in this study in response to a rockcod supports the findings of Ramasamy *et al*. (2015) on another damselfish, the spiny chromis (*Acanthochromis polyacanthus*), which showed substantially improved fast-start performance when they had been exposed to olfactory or visual cues from a common predator, the dusky dottyback (*Pseudochromis fuscus*). This is only the second time such an effect has been demonstrated and so still requires further study to conclude that this forewarning response is a general phenomenon. However, it is likely that this is a general effect among fishes and one of a number of factors that affect fast-start performance, which often makes the response context dependent (Domenici, 2010). Other studies have shown that a range of recent experiences can affect the fast-start response of fishes. These can be divided into environmental effects that directly affect the physical capacity of the responding prey to react quickly, such as water temperature, low pH and pollutants (McKenzie *et al*., 2007; Allan *et al.,* 2013, 2015), and those that work through behavioural modulation (for review see Domenici, 2009). The present study is an example of the latter.

Our results indicate that being forewarned of a threat leads to a more effective fast-start response. Not surprisingly, studies have shown that those fishes that have a well-developed fast-start response survive better in direct encounters with predators (Walker *et al*., 2005; but see Holmes and McCormick, 2009). The more effective fast start once forewarned of the presence of a common rockcod predator suggests that physiological and/or behavioural priming of an escape response is taking place to reduce the decision time of whether or not to initiate a burst, as indicated here by the reduced latency. These results suggest that some neural processing (i.e. facilitation) may be occurring in the brain region responsible for processing the output of the large Mauthner motor neurons that are usually responsible for the rapid onset of the fast-start response (Domenici and Blake, 1997). It is likely that this forewarning of a predator leads to an elevation of blood cortisol (Oliveira *et al*., 2014), which mobilizes glucose (Barton, 2002), heightens neurological activity and speeds decisions on simple or well-rehearsed tasks (Mendl, 1999; Akinola and Mendes, 2012; Sandi, 2013). Through this mechanism, moderate levels of cortisol should enhance vigilance (Barreto *et al*., 2014). Experiments on humans also show that glucose elevation enhances spatial memory and recognition speed (Owen *et al*., 2013). The physiological systems typically activated in response to a stressor (i.e. the sympathetic nervous system, the hypothalamic–pituitary–adrenocortical axis and the central neurotransmitter and neuropeptide systems) all have effector mechanisms in brain circuits that play central roles in information processing (Sandi, 2013). Our study found that latency was significantly reduced when fish had been exposed to rockcod cues prior to being startled, in keeping with the priming action of a cortisol response. This cortisol priming may also allow the oxygen debt from an anaerobically fuelled burst to be repaid quickly because of the enhanced availability of blood glucose, thereby minimizing any reduced performance when multiple fast starts are required in quick succession. It is likely that at least some of the forewarning effect evidenced in the present study is attributable to the cognitive effects of glutocorticoids on information processing, although the exact mechanism requires further study.

The miscategorization of lionfish as a non-predator is not specific to our study species. A closely related species, the blue chromis (*Chromis viridis*), failed to exhibit antipredator behaviours in the presence of the red lionfish, leading to an increase in mortality rates (Lönnstedt and McCormick, 2013). This occurred, as in the present study, despite being taught that the sight and smell of the lionfish represented a predatory threat through associative learning. The lack of a forewarning effect for lionfish is most probably because of their ability to circumvent being identified as a predator. There is no reason why this ability should be specific to the genus Pomacentridae, and it is likely that lionfish are variously misclassified as non-predators by many of their potential prey species, although genus-level differences in response to lionfish remain to be studied.

The lack of a forewarning effect from lionfish to the whitetail damselfish prey is surprising given the highly evolved mechanisms for rapidly labelling novel predators as threats among fishes and many invertebrates. Learned recognition may arise through mechanisms that include direct interactions with a predator, the detection of predator odour coupled with a chemical alarm cue (associative learning), or detection of cues released following the digestion of consumed conspecifics (Ferrari *et al*., 2010). Moreover, fishes have been shown to have a sophisticated mechanism of reinforcing some risk associations, de-emphasizing (‘forgetting’) others (Mitchell *et al*., 2011a) and transferring this information to secondary individuals that have not been in direct threat with the potential predator (social learning; Crane and Ferrari, 2013; Manassa *et al*., 2013). These mechanisms are so strongly developed, ubiquitous and non-discriminatory that fishes have been trained to be threatened by such novel stimuli as a red light (Yunker *et al*., 1999), a black disc (Wisenden and Harter, 2001) or lemon juice (Leduc *et al*., 2007). Research on tropical fish species suggests that only a single learning opportunity is required for prey to catalogue a threat, which means that the identity of a novel threat is rapidly disseminated throughout the local prey population (Mitchell *et al*., 2011b). For juvenile fishes that have just transitioned from the larval phase and are effectively naïve to reef-based predators, these mechanisms have been shown to be very rapid and vital for survival in the field (Lönnstedt *et al*., 2012; Manassa and McCormick, 2013). Juvenile fish that had not learnt common predators through these mechanisms died five to eight times faster in these studies. Thus, there is a large body of research indicating that prey fish should be able to identify the threat status of novel predators rapidly, regardless of whether they are native or invasive. What is perplexing is how lionfish manage to evade these mechanisms of threat labelling.

Olfaction is a key sense for chemical associative learning of new threats in aquatic animals through the co-occurrence of a relevant chemical alarm cue with an olfactory or visual signal. Lionfish may circumvent forewarning of prey through olfaction in three non-exclusive ways: masking of scent (seen previously in some insects, e.g. Chivers and Smith, 1998); a lack of scent (which is unlikely because aquatic predators leak kairomones that act as warning cues; Ferrari *et al*., 2010); or a modification of scent (alarm cue or threat odour, as has been shown for the environmental smells modifying chemical alarm cues; Lönnstedt *et al*., 2013). The likelihood of these mechanisms of crypsis are discussed by Lönnstedt and McCormick (2013), but these are speculative and await further study.

The present study supports the hypothesis that lionfish can circumvent common mechanisms of predator labelling and are effectively treated by prey as non-predators in laboratory trials. This enables them not to trigger the forewarning effect that enhances the efficacy of the fast-start response elicited by prey in response to a strike. This avoidance of a forewarning effect joins the many other behavioural mechanisms used by lionfish that enable them to achieve one of the highest successful strike rates recorded for any fish (Green *et al*., 2011). Other mechanisms include producing oral currents to orient prey to their mouths (Albins and Lyons, 2012), cooperative hunting (Lönnstedt *et al*., 2014), prey herding (Fishelson, 1997) and visual distraction by fin waving (Fishelson, 1975). Further studies are required to determine variability among prey species in their ability to label lionfish appropriately as predators and, in doing so, use appropriate antipredatory behaviours to mitigate lionfish predation.
